# Repeat stereotactic radiosurgery for progressive vestibular schwannomas after previous radiosurgery: a systematic review and meta-analysis

**DOI:** 10.1007/s10143-021-01528-y

**Published:** 2021-04-13

**Authors:** Anne Balossier, Jean Régis, Nicolas Reyns, Pierre-Hugues Roche, Roy Thomas Daniel, Mercy George, Mohamed Faouzi, Marc Levivier, Constantin Tuleasca

**Affiliations:** 1 Functional, and Stereotactic Neurosurgery Service and Gamma Knife Unit, Assistance Publique - Hôpitaux de Marseille, Timone Hospital, Marseille, France; 2 Aix-Marseille University, Inserm, INS, Inst Neurosci Syst, Marseille, France; 3 University of Lille, Inserm, CHU de Lille, U1189 - ONCO-THAI –Laser Assisted Therapies and Immunotherapies for Oncology, F-59000 Lille, France; 4 Neurosurgery and Neurooncology Department, CHU de Lille, F-59000 Lille, France; 5 Neurosurgery Service, Neurochirurgie Hôpital Nord, Pôle NEUROSCIENCES, Hôpital Nord, Marseille, France; 6 Department of Clinical Neurosciences, Neurosurgery Service and Gamma Knife Center, Centre Hospitalier Universitaire Vaudois (CHUV), Lausanne, Switzerland; 7 Faculty of Biology and Medicine (FBM), University of Lausanne (Unil), Lausanne, Switzerland; 8 Department of Otorhinolaryngology, Head & Neck Surgery, Centre Hospitalier Universitaire Vaudois (CHUV), Lausanne, Switzerland; 9 Division of Biostatistics, Center for Primary Care and Public Health (Unisanté), Université de Lausanne, Lausanne, Switzerland; 10 Signal Processing Laboratory (LTS 5), École Polytechnique Fédérale de Lausanne (EPFL), Lausanne, Switzerland

**Keywords:** Stereotactic radiosurgery, Vestibular schwannoma, Gamma Knife, Facial nerve, Cochlear nerve, Hearing

## Abstract

Vestibular schwannomas (VS) are slow-growing intracranial extraaxial benign tumors, developing from the vestibular part of the eight cranial nerves. Stereotactic radiosurgery (SRS) has now a long-term scientific track record as first intention treatment for small- to medium-sized VS. Though its success rate is very high, SRS for VS might fail to control tumor growth in some cases. However, the literature on repeat SRS after previously failed SRS remains scarce and reported in a low number of series with a limited number of cases. Here, we aimed at performing a systematic review and meta-analysis of the literature on repeat SRS for VS. Using PRISMA guidelines, we reviewed manuscripts published between January 1990 and October 2020 and referenced in PubMed. Tumor control and cranial nerve outcomes were evaluated with separate meta-analyses. Eight studies comprising 194 patients were included. The overall rate of patients treated in repeat SRS series as per overall series with first SRS was 2.2% (range 1.2–3.2%, *p* < 0.001). The mean time between first and second SRS was 50.7 months (median 51, range 44–64). The median marginal dose prescribed at first SRS was 12 Gy (range 8–24) and at second SRS was 12 Gy (range 9.8–19). After repeat SRS, tumor stability was reported in 61/194 patients, i.e., a rate of 29.6% (range 20.2–39%, I^2^ = 49.1%, *p* < 0.001). Tumor decrease was reported in 83/194 patients, i.e., a rate of 54.4% (range 33.7–75.1%, I^2^ = 89.1%, *p* < 0.001). Tumor progression was reported in 50/188 patients, i.e., a rate of 16.1% (range 2.5–29.7%, I^2^ = 87.1%, *p* = 0.02), rarely managed surgically. New trigeminal numbness was reported in 27/170 patients, i.e., a rate of 9.9% (range 1.4–18.3%, *p* < 0.02). New facial nerve palsy of worsened of previous was reported in 8/183 patients, i.e., a rate of 4.3% (range 1.4–7.2%, *p* = 0.004). Hearing loss was reported in 12/22 patients, i.e., a rate of 54.3% (range 24.8–83.8%, I^2^ = 70.7%, *p* < 0.001). Repeat SRS after previously failed SRS for VS is associated with high tumor control rates. Cranial nerve outcomes remain favorable, particularly for facial nerve. The rate of hearing loss appears similar to the one related to first SRS.

## Introduction

Vestibular schwannomas (VS) are intracranial extraaxial benign tumors, slow growing, developing from the vestibular part of the eight cranial nerves [47]. They account for approximately 10% of primary brain tumors [57]. The relative incidence is 0.6–0.8 per 100,000 individuals per year [47]. Due to the increased use of advanced radiographic imaging, and in particular contrast-enhanced magnetic resonance imaging (MRI), more and more VS are diagnosed incidentally in patients without any symptom. The most common symptom is unilateral hearing decline, followed by tinnitus and vertigo [19]. Classically, VS are frequently diagnosed around the fifth decade of life. Most of the authors report female preponderance [39]. It has been recently acknowledged that three different growth patterns are commonly described, including no/very slow growth, slow growth (2 mm/year), or fast growth (> 8 mm/year) [21]. Such variety of tumor growth rates and interventional outcomes, including for incidentally discovered lesions, make long-term management a matter of debate [46].

The current gold standard for diagnosis is thin-cut axial MRI of the head with contrast enhancement (gadolinium) [4]. Recent evidence suggested that non-enhanced thin-slice T2-weighted MRI might be sufficient for initial screening [5]. The advantages of such approach include not only reduced risk of adverse reactions, but also reduced cost [4]. Moreover, this offers also a high contrast between the VS, cerebrospinal fluid, and the adjacent structures [36]. The T2 CISS is also considered highly sensitive and specific in detecting small VS, close to the T1 gadolinium weighted. The MRI allows evaluating volumetric course during time. Various measurements have been applied, including mm/year-based model, cm^3^/year-based model, and a volume-doubling time (VDT)-based model. Some authors suggested that VDT-based model was the most accurate in describing VS growth [56].

Newly diagnosed VS can benefit from “wait and scan” strategy [21], stereotactic radiosurgery (SRS) [28, 43, 52], radiotherapy [51], or microsurgical resection [6, 48, 55]. Recurrent or progressive residual VS might be treated by SRS [15] or microsurgical resection [38] depending on tumor volume and patients’ specific characteristics (age, medical comorbidities) [49]. In the opinion of some authors, microsurgery remains the most prevalent strategy, which is particularly applicable to large VS. However, during the recent years, there has been an increasing trend towards “wait-and-scan” [22] and SRS, the former especially for small- to medium-sized tumors, or in the frame of combined approaches, following planned subtotal resection [6, 16].

Stereotactic radiosurgery is beginning to have a long-term scientific track record as first intention treatment for small- to medium-sized VS, with high tumor control rates and low morbidity rates. In case of failure, which can occasionally occur, microsurgical resection is generally advocated. Recently, there is a growing literature with regard to the role of repeat SRS for growing VS after the first failed SRS treatment [11, 15]. Thus, some authors advocated for the use of a second SRS instead of microsurgical resection, whenever possible, alone or in combination with the former, in the frame of combined approaches [55]. However, the total number of patients reported is quite low, and the exact safety and efficacy are not widely accepted. Moreover, the exact indications might vary among centers. Thus, the literature on this topic is scarce.

Here, we performed systematic review and meta-analysis of series reporting retreatment by SRS for growing VS after previously failed SRS. We have chosen this topic as, up-to-date, the literature is scarce and includes a limited number of series. Moreover, the number of patients included in the reported series is low. There are several pending questions related to the role of retreatment by SRS (after first SRS) of VS including the timing of second SRS, the tumor control, and the potential induced neuropathies (including hearing preservation) as well as how to manage radiation-induced changes, related both to tumor swelling with further pseudoprogression appearance. Thus, we describe tumor control rates, as well as cranial nerve outcomes (whenever reported), with complications and their relative incidence. We present the indications for such retreatment, as depicted in the current literature.

## Methods

### Article selection and data extraction

A PubMed search was performed for entries between January 1990 and October 2020 using the following query guidelines: ((vestibular AND (radiosurgery OR Gamma Knife)) AND (schwannoma) OR (retreatment)). Inclusion criteria were as follows: peer-reviewed clinical study or case series of VS retreated with SRS, independently of the device, clearly specified and presented as independent series of second SRS. Excluded were as follows: abstract, case reports, non-English studies, conference papers and series where fractionated radiotherapy was used.

Eight studies comprising 194 patients were included [7, 9, 11, 15, 18, 26, 27, 61]. The detailed study characteristics can be seen in Tables 1, 2, and 3. There were only Gamma Knife (GK, Elekta Instruments, AB, Sweden) studies including reports of second SRS after a first failed SRS. No Linear Accelerator (Linac) or Cyberknife studies specifically reported outcomes following retreatment, in the frame of a separate clinical article.

**Table 1:** Basic demographic data

Series	Number	Age, years Mean/median (range)	Follow-up after second GK, median	Marginal dose (first GK, Gy) Median, range	Marginal dose (second GK, Gy) Median, range	Time between GK (months, range)	Initial size (mm)/volume (mL) (median, range)	Posttreatment I size (median, range)	Posttreatment II size (median, range)
Dewan et al. (2008)	11	Median 63 (49–78)	-	12 (11–13.2)	12 (11–13.2)	Mean 51 (24–136)	16.9 mm (9–30.6)	20.9 mm (13.5–33.4)	18.4 mm (12–31.2)
Yomo et al. (2008)	8/1951 (0.4%)	Median 52 (41–76)	64 (26–121)	12 (12–14)	12 (10–12)	Median 46 (35–99)	0.51 mL (0.1–1.4)	1.28 mL (0.54–3.07)	-
Liscak et al. (2009)	26/351 (7.4%)	Median 56 (21–80)	43 (22–121)	12.5 (8–24)	13 (12–19)	Median 43 (12–123)	1.1 mL (0.2–3.8)	2.9 mL (0.7–6.5)	-
Kano et al. (2010)	6/1352 (0.4%)	Median 51 (44–77)	29 (13–71)	13 (12.5–18)	11 (10–12.5)	Median 63 (25–169)	0.5 mL (0.08–2.3)	2.1 mL (0.9–6.4)	1.9 mL (0.6–5.2)
Lonneville et al. (2015)	25/728 (3.4%, 27 procedures)	Median 53 (32–82)	Mean 46 (24–110)	12 (12–13)	12 (11–15)	Median 45 (24–112)	0.9 mL (0.1–9.2)	2.3 mL (0.2–8.3)	-
Fu et al. (2018)	28/1156 (2.4%, 38 of whom10 combined approach)	Mean 57 (42–81)	66 (13–129)	11 (10.4–12.3)	11.8 (9.8–13)	Median 52 (19–86)	1.7 mL (0.072–7.8)	2.9 mL (0.27–9.4)	2.1 mL (0.1–9.4)
Iorio-Morin et al. (2018)	76	-	51.7 (3.7–228)	12.5 (10–18)	12 (12–17)	Median 54 (12–185)	1.2 mL (0.08–17.9)	3.1 mL (0.4–19.5)	-
Hafez et al. (2020)	14/560 (2.5%)	Median 51 (26–69)	60 (24–144)	12 (12–17)	12 (13–17)	Median 44 (24–96)	2.4 mL (0.27–3.8)	3.8 mL (1.2–7.6)	-

**Table 2:** Tumor control, trigeminal, and facial nerve outcomes

	Tumor stability	Tumor decrease	Tumor increase	Trigeminal nerve-numbness (new)	Trigeminal nerve, paresthesia (new)	Trigeminal nerve-pain (new)	Trigeminal nerve (improved)	Facial nerve (stable)	Facial nerve (new/worsened)	Hemifacial spasm
Dewan et al. (2008)	1/11 (9%)	8/11 (73%)	2/11 (18%)	2/11 (18%)	-	-	Facial numbness 1/11 (9%)	11/11 (100%) Pre-SRS HB I 9/11, II 2/11	-	-
Yomo et al. (2008)	2/8 (25%)	6/8 (75%)	0/8 (0%)	-	-	-	-	8/8 (100%)	-	-
Liscak et al. (2009)	7/24 (29.2%)	15/24 (62.5%)	2/24 (8%)	-	Mild paresthesia 2/24 (8%) 6 and 20 months after	-	-	-	1/24 (4.2%) HB 2 became 3 5 months after	3/24 (12.5%)
Kano et al. (2010)	2/6 (33.3%)	4/6 (66.7%)	-	0/6 (0%)	-	-	0/6 (0%)	0/6 (0%)	0/6 (0%)	-
Lonneville et al. (2015)	8/27 (29.6%)	15/27 (55.5%)	4/27 (14.8%)	0/27 (0%)	-	-	0/27 (0%)	0/27 (0%)	0/27 (0%)	-
Fu et al. (2018)	7/28 (25%)	19/28 (67.8%)	2/28 (7.1%)	4/28, of whom 2/28 (7.1%) definitive	-	-	-	-	4/28 of whom 2/28 (7.1%) definitive	9/28, of whom 5/28 (17.8%) definitive
Iorio-Morin et al. (2018)	25/76 (34%)	12/76 (16%)	39/76 (50%)	23/76 (30%)	-	Pain 5/76 (7%)	-	70/76 (92.1%)	6/76 (7.9%) 5/76 (6.6%) considered specific for SRS	-
Hafez et al. (2020)	9/14 (64.3%)	4/14 (28.6%)	1/14 (7.1%)	0/14 (0%)	1/14 (7.1%)	-	0/14 (0%)	0/14 (0%)	0/14 (0%)	0/14 (0%)

**Table 3:** Vestibular and cochlear nerve outcomes, TTE, ARE, and other complications, further treatment (when reported)

	Vestibular	Cochlear (worsened)	Transient tumor expansion	Adverse radiation events (ARE)	Other complications	Further surgery
Dewan et al. (2008)	-	1/1 (100%) (10 no prior useful hearing) Gardner I to II	-	2/11 (slight transient peduncular edema)	-	-
Yomo et al. (2008)	-	2/3 (66.6%) (5 no prior useful hearing)		-	-	-
Liscak et al. (2009)	1/24 (4.2%)	0/2 (0%)	-	-	1 ventriculo-peritoneal shunt (22 months later)	1/24
Kano et al. (2010)	-	0 (0%)	-	1/6 (slight transient peduncular edema) 16 months after GK	-	-
Lonneville et al. (2015)	-	5/5 (100%)	12/28 (42.8%)	-	-	-
Fu et al. (2018)	-	1/2 (50%)	12/28	1/28 (3.6%) transient peduncular edema being hospitalized (dexamethasone and manitol)	-	-
Iorio-Morin et al. (2018)	41/76 (55%) imbalance	1/6 (16.7%)	13/76 (17.5%) after median of 12.5 months (3–24)	-	-	8/76 (10.5%) 3 for tumor control, 5 for symptom control (facial pain, imbalance, vertigo)
Hafez et al. (2020)	-	2/3 (66.6%)	-	0/14 (0%)	-	-

**Fig. 1:**
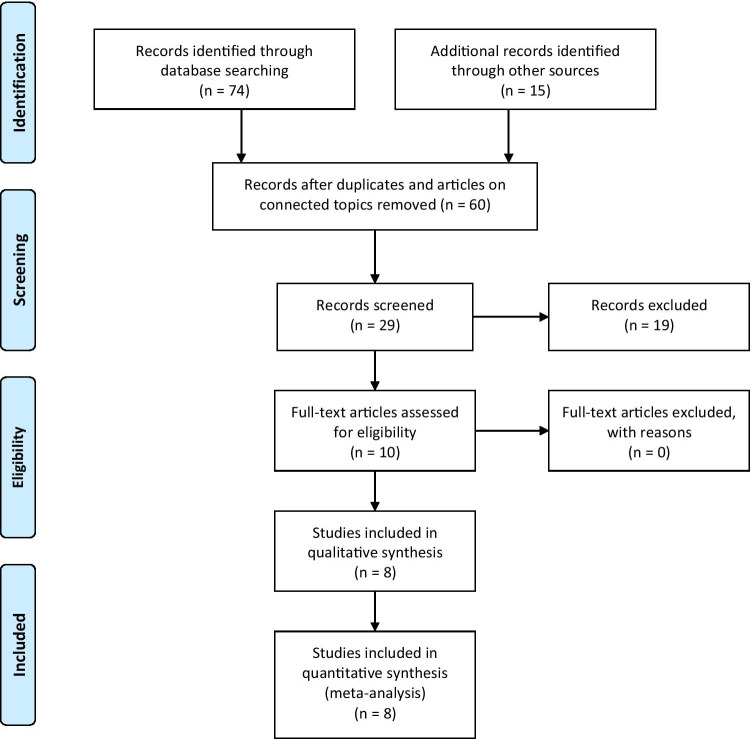
Prisma flowchart with study selection details

The article selection is collated in Fig. 1. Two separate reviewers (AB, CT) applied the inclusion criteria to the PubMed search result; there were no disagreements.

This study was performed in accordance with the published Preferred Reporting Items for Systematic Reviews and Meta-Analyses (PRISMA) guidelines [33].

Data extraction was performed as per individual study, while paying special attention to tumor control aspects (stability, decrease, increase) and cranial nerve outcomes (pertaining to trigeminal, facial, and/or cochlear and vestibular nerves).

### Indication for retreatment

Treatment failure has been heterogeneously defined among studies, usually as continuous growth requiring subsequent intervention after a minimum 2 years of follow-up [61]. Some authors classified the response to GK as regression (more than 10% volume reduction), stabilization (volume variation within 10%), enlargement (more than 10% volume increase not requiring further intervention), and failure (uncontrollable tumor growth requiring further intervention and/or appearance of disabling radiation side effect) [61].

### Specific outcome measurements

The House-Brackmann scale was used to asses facial nerve function [14]. The Gardner-Robertson scale was used to assess hearing function, defined as functional hearing for classes I and II [10].

### Adverse radiation events

Magnetic resonance imaging (MRI) was used to assess presence of edema at cerebellar and/or brainstem level. The most frequently used sequences were T2 w (CISS) and FLAIR.

### Statistical analysis using OpenMeta (Analyst) and random-effects model

Due to the high variation in study characteristics, a statistical analysis using a binary random-effects model (DerSimonian-Laird method) was performed. We used OpenMeta (analyst) software from the Agency for Healthcare Research and Quality.

Weighted summary rates were determined using meta-analytical models. Testing for heterogeneity was performed for each meta-analysis.

Pooled estimates using meta-analytical techniques were obtained for all the outcomes previously described in the same section.

## Results

### Number of patients in repeat SRS series as per overall series with first SRS

**Fig. 2:**
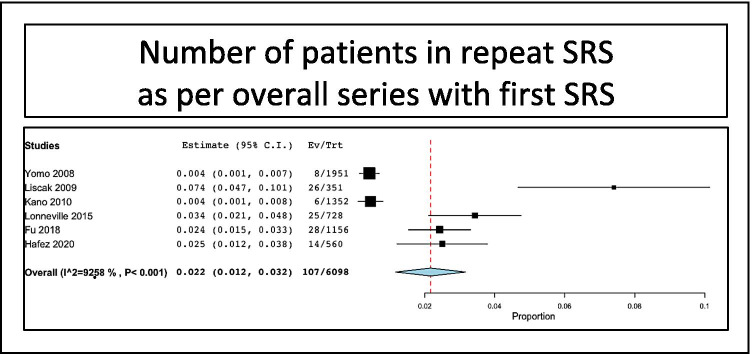
Number of patients in repeat SRS series as per overall series with first SRS

The overall rate of patients treated in repeat SRS series as per overall series with first SRS was 2.2% (range 1.2–3.2%, I^2^ = 92.5%, *p* heterogeneity < 0.001, *p* < 0.001) (Fig. 2).

### Time between first and second SRS

The mean time between first and second SRS was 50.7 months (median 51, range 44–64). The minimal and maximal time frames were 12 and 185 months, respectively.

### Marginal dose prescription

The median marginal dose prescribed at first and second SRS were 12 Gy (range 8–24) and 12 Gy (range 9.8–19), respectively.

### Tumor control after repeat SRS

**Fig. 3:**
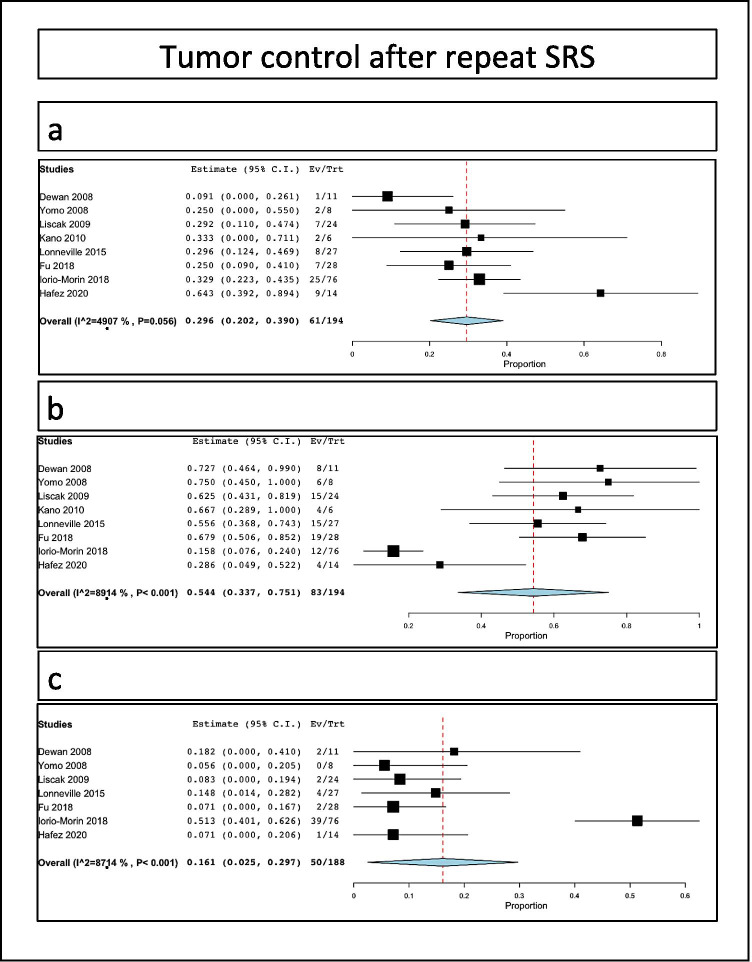
Tumor control rates after SRS for VS: **a** stability; **b** tumor; **c** progression rates

Tumor stability after retreatment was reported in 61/194 patients, i.e., a rate of 29.6% (range 20.2–39%, I^2^ = 49.1%, *p* heterogeneity 0.05, *p* < 0.001; Fig. 3a).

Tumor decrease after retreatment was reported in 83/194 patients, i.e., a rate of 54.4% (range 33.7–75.1%, I^2^ = 89.1%, *p* heterogeneity *p* < 0.001, *p* < 0.001; Fig. 3b).

Tumor progression after retreatment was reported in 50/188 patients, i.e., a rate of 16.1% (range 2.5–29.7%, I^2^ = 87.1%, *p* heterogeneity *p* < 0.001, *p* = 0.02; Fig. 3c).

### Transient tumor expansion after SRS

Transient tumor expansion after SRS was specifically reported in two series [15, 27], ranging between 17.5 and 42.8%.

### Cranial nerve outcomes after repeat SRS

**Fig. 4:**
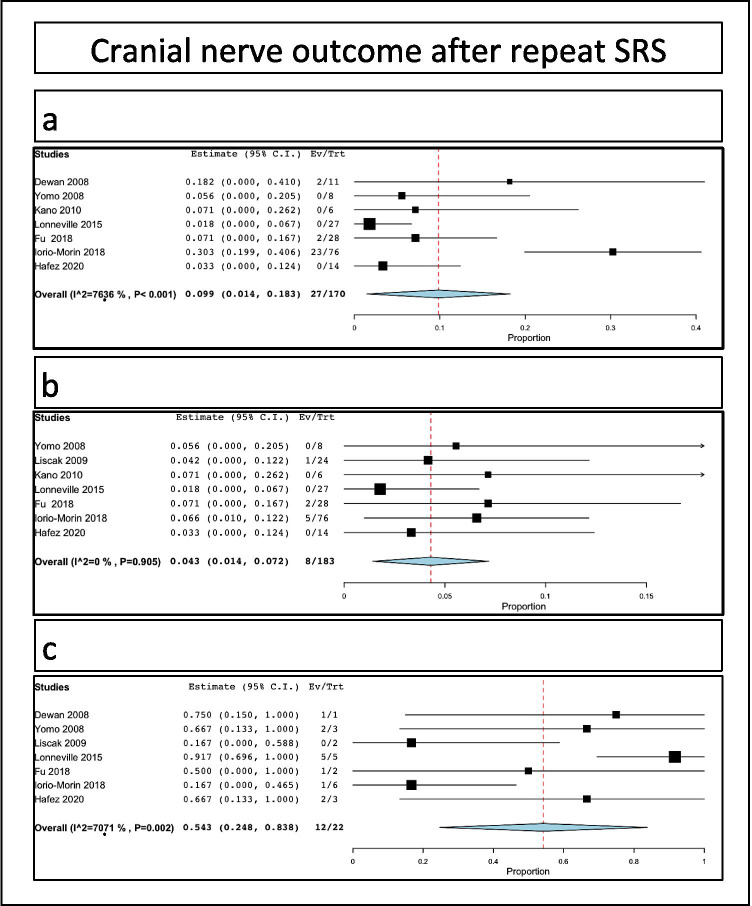
Specific cranial nerve outcomes after SRS: **a** trigeminal; **b** facial; **c** cochlear

New trigeminal numbness after retreatment was reported in 27/170 patients, i.e., a rate of 9.9% (range 1.4–18.3%, I^2^ = 76.36%, *p* heterogeneity *p* < 0.001, *p* = 0.02; Fig. 4a).

New facial nerve palsy of worsened facial nerve outcome of previously existing one was reported in 8/183 patients, i.e., a rate of 4.3% (range 1.4–7.2%, I^2^ = 0%, *p* heterogeneity *p* = 0.905, *p* = 0.004; Fig. 4b).

Hearing loss after retreatment was reported in 12/22 patients, i.e., a rate of 54.3% (range 24.8–83.8%, I^2^ = 70.7%, *p* heterogeneity *p* = 0.002, *p* < 0.001; Fig. 4c).

### Ventriculo-peritoneal shunt

One series [26] reported the necessity of a ventriculo-peritoneal shunt 22 months after second SRS in one case.

## Discussion

The results of our systematic review and meta-analysis show that repeat SRS for VS remains safe and effective, as the results after a first SRS [17]. Here, we included 8 studies comprising 194 patients, all treated with GK as second SRS after a previously failed SRS for VS. We report overall tumor stability in 29.6% of cases, decrease in 54.4%, and further tumor progression in 16.1%. However, only a small number of cases with further progression underwent surgical resection in the reported series. This is most probably related to pseudoprogression phenomenon, although this was not clearly stated. Moreover, such pseudoprogression appeared to be as high as 42% in one of the included studies. The rate of new trigeminal neuropathy was 9.9%. The rate of facial nerve palsy appearance or worsening was 4.3%. Of note, after first SRS, trigeminal preservation rates vary between 74 and 99% [8, 12, 21, 24, 31, 41, 44, 55] and facial preservation ranges between 84 and 100% [8, 12, 21, 24, 31, 41, 44, 55]. In a recent meta-analysis (which included 45 articles and 4234 patients), the crude rate of hearing preservation after first SRS was 51% at a median follow-up period of 44 + / − 32 months [60]. In the present meta-analysis, the rate of hearing loss was 54.3% going up to as high as 83.8%. The rate of hearing loss after second SRS appears similar to the one after first SRS.

The largest series included in the present meta-analysis was the one of Iorio-Morin et al. [15] which analyzed 76 patients. The authors reported high actuarial tumor control rates at 2, 5, and 10 years following SRS of 98.6%, 92.2%, and 92.2%, respectively. Worsening of the facial nerve function attained 7%. In another series, Fu et al. [9] reported 100% tumor control after second SRS after a median follow-up period of 75 months. Volumetric tumor response after the second procedure could not be predicted by the volumetric response after first SRS. Thus, the authors concluded that this justifies considering repeat SRS even for tumors that did not show any volumetric response and displayed continuous growth after first treatment. Moreover, there is an increased risk of mild facial and trigeminal nerve dysfunction after second as compared to first SRS. Such results fairly compared to what is reported here in our meta-analysis.

The timing for retreatment after first SRS failure for VS has been controversial in the current literature. Retreatment indication for microsurgery or SRS is usually related to tumor (volume, edema) or patient (age, symptoms, medical comorbidities) specific factors. With regard to tumor-related aspects, one should keep also in mind the probability of transient tumor expansion (TTE) [42], which varies between 17 and 74% of patients after first GK [13, 35, 62]. Classically, TTE typically occurs between 3 and 9 months after first GK treatment, with a peak at 6 months, induced by internal swelling [34]. This should not be confounded with real tumor progression. Subsequent volumetric measurement is the key to evaluate such progression, while some authors suggested even preradiosurgical radiomics [59]. One should keep in mind that at least 2 years follow-up is usually required [23], avoiding misjudging any temporary tumoral swelling [32]. This should be further judged in the context of presence or absence of patient’s symptoms. Additional neuroimaging assessment (other than MRI) might be also useful. In a series by Lonneville et al. [27], the authors evaluated the role of PET during follow-up course, which showed a significant metabolic decrease of the tumor, further considered as TTE. Moreover, one other particular aspect is related to potential cystic components or entrapment cysts [29], the latter with much-complicated decision making, depending on the clinical context [53]. With regard to patient-related aspects, indications for additional management after initial GK might include trigeminal neuropathy (numbness, trigeminal pain, facial neuropathy), facial neuropathy (palsy, hemifacial spasm [18]), or vestibular (major imbalance).

In line with the previous, the definition of treatment failure after first SRS is not always straightforward. In our opinion, several key aspects should be considered before concluding treatment failure: duration of at least 2 years after first SRS (preferably even 3–4 years, unless symptomatic mass effect) and minimum 3 time points of follow-up MRI displaying continuous growth. Further decision to retreat should be delayed in absence of symptomatic mass effect. Transient tumor expansion should always be kept in mind, and is frequently associated with transient loss of central contrast enhancement. Clinically relevant tumor progression should be certainly differentiated from TTE and should rule the therapeutic decision. The neurosurgical team involved in decision making should be aware of classical changes after SRS.

Following primary SRS, 5-year tumor control rate is 90–98% [1, 3, 28]. The key factor in retreating VSs with SRS is to formally consider that first SRS was a failure. In some series [11], the indication for retreatment was small- to medium-sized VS, less than 30 mm in diameter with documented tumor growth. While a vast majority of authors agree to irradiation of the whole VS during second SRS, some series advocated to irradiate only the progressive part of the tumor [27]. In our opinion, such an approach is not taking into account the radiobiological aspects [54], which might not consider several aspects pertinent to the tumor itself. Patients with a VS needing repeat treatment after initial SRS are a selected group of patients, with somehow unfavorable tumor response due to SRS and, most probably, a different radiobiological response. Another strategy, presented in the same study, was to perform even third irradiation for tumor growth [27]. However, such strategy might engender further TTE and additional symptoms. Advantages of SRS as preferred treatment modality in this indication are also related to avoid risks of open microsurgical resection, including meningitis (1–3%) [45], hydrocephalus [40], or cerebro-spinal fluid leakage [2].

A key factor for tumor control is the marginal dose to be prescribed. Both in the literature and in our experience, we do not favor prescribing marginal doses of less than 11 Gy at the time of first or second GK. As illustrated here, some of the patients received even doses of less than 10 Gy, which might explain failures of first and/or second SRS [20]

Here, we reported rates up to 10% of trigeminal nerve neuropathy. Such results should take into account the related volume of VS (close to the trigeminal nerve or in contact with the former). Also, dosimetric aspects are highly important, such as the location of the trigeminal root entry zone (REZ) in relationship with parts of the VS, REZ which should be visualized and excluded from the prescription isodose line, if possible. Another aspect is related to the interface with the brainstem, which should receive the steepest gradient during treatment planning [37]. Facial nerve neuropathy remains rare after repeat SRS, with an overall rate of 4.2%, similar to that of first SRS. Hearing preservation is probably the most challenging aspect after second GK. The dose to the cochlea has been now standardly reported for first SRS [30, 50], but scarcely after the second one.

The effects of radiosurgery on the cochlear nerve in the literature have been frequently attributed to user technique. It has been previously acknowledged that the maximal dose received by the cochlea after a first SRS might play a role in hearing decline during follow-up course [50]. The data of cumulative cochlear dose (at first and second SRS, respectively) remain scarce and are frequently not reported. An additional aspect is that most users measure such dose only at the level of the modulus itself. Linskey et al. [25] suggested that the basal turn of the cochlea should also receive the lowest possible dose (ideally less than 4–5.3 Gy). Moreover, other authors advocated for a role of radiation dose rate, especially in the appearance of clinical acute and subacute effects after first SRS [52].

No patient developed radiation-induced tumors [58].

Future directions of clinical research shall include frequency of pseudoprogression phenomenon with the exact timing, MRI aspect, more clear definition of failure (clinical and/or radiological), and strong reliable markers of failure, including on neuroimaging.

Our meta-analysis has several inherent limitations. One limitation is related to patients, which previously received also another type of radiation. In this sense, only one patient included in a study [7] was previously treated with proton beam therapy (inside a series of 11 patients). However, in that series, the specific outcomes were not detailed as per patient. A second limitation is related to the minimal tumor coverage at the time of first SRS which varies depending on studies, as some authors reported a minimal cutoff of 90% [9]. This could have further influenced tumor control. A third limitation might be related to previously used treatment paradigms. Some series included, beside repeat GK after initial GK, a second GK after initial combined approach (subtotal microsurgical resection followed by SRS) [9]. Such tumors benefiting from previous microsurgical resection might have had a different radiobiology, although such aspect remains purely theoretical.

## Conclusion

Repeat SRS after previously failed SRS for VS is associated with high tumor control rates. Tumor progression was reported in an overall rate of 16%, while only some series reported further necessity of surgical management. Thus, in case of such progression, one should exclude a TTE. Cranial nerve outcomes remain favorable, particularly concerning the facial nerve. Hearing loss rates are similar to first SRS.

## Data Availability

Data is presented in tables and figures, as it is a systematic review.

## Code availability

N/A
